# Effect of a Tailored eHealth Physical Activity Intervention on Physical Activity and Depression During Postpartum: Randomized Controlled Trial (The Postpartum Wellness Study)

**DOI:** 10.2196/64507

**Published:** 2025-05-22

**Authors:** Sylvia E Badon, Nina Oberman, Maya Ramsey, Charles P Quesenberry, Elaine Kurtovich, Lizeth Gomez Chavez, Susan D Brown, Cheryl L Albright, Mibhali Bhalala, Lyndsay A Avalos

**Affiliations:** 1 Division of Research, Kaiser Permanente Northern California, 4480 Hacienda Dr, Pleasanton, CA, 94588, United States, 1 4152646944; 2 School of Medicine, University of California Davis, Davis, CA, United States; 3 University of Hawaii at Manoa, Honolulu, HI, United States; 4 Redwood City Medical Center, Kaiser Permanente Northern California, Redwood City, CA, United States

**Keywords:** eHealth, depression, mental health, physical activity, postpartum, postpartum depression, randomized controlled trial, RCT, electronic health, intervention, effectiveness, depressive symptoms, California, health care, wrist-worn accelerometer, wearables, accelerometer, intent-to-treat, linear regression

## Abstract

**Background:**

Strong evidence suggests physical activity (PA) can ameliorate postpartum depression (PPD) symptoms; however, many postpartum individuals do not meet PA guidelines. Electronic health (eHealth) interventions are a promising approach to address common barriers to PA during postpartum.

**Objective:**

To test the effectiveness of a tailored eHealth PA intervention for increasing PA and decreasing depressive symptoms in individuals at high risk for PPD.

**Methods:**

We conducted a randomized controlled trial within the Kaiser Permanente Northern California integrated health care delivery system. From November 2020 to September 2022, individuals 2‐6 months postpartum at high risk for PPD were randomized to an eHealth PA intervention (n=50) or usual care (n=49). The eHealth PA intervention group received access to an online library of 98 ten-minute workout videos developed for postpartum individuals, including interaction with their infants. At baseline, 3 months, and 6 months postrandomization, surveys were used to assess depressive symptoms, PA, sleep quality, anxiety symptoms, perceived stress, and mother-infant bonding. PA was also measured using a wrist-worn accelerometer for 7 days at each timepoint. Primary outcomes were depressive symptoms and device-measured moderate-to-vigorous intensity PA (dm-MVPA) at 3 months postrandomization. Secondary outcomes were self-reported MVPA (sr-MVPA) at 3 and 6 months postrandomization and depressive symptoms and dm-MVPA at 6 months postrandomization. Intent-to-treat and modified intent-to-treat (excluding participants in the intervention group who did not watch at least 1 video) analyses were conducted using linear regression adjusted for variables used in the randomization procedure and using multiple imputation to account for missing data.

**Results:**

Participants were 4 months postpartum at baseline with moderately severe depressive symptoms (mean PHQ-8 [Patient Health Questionnaire-8] score=12.6), on average. Intent-to-treat analyses indicated no association between the intervention and change in depressive symptoms (mean difference=−0.9; 95% CI −3.3 to 1.5), dm-MVPA per day (mean difference=−4.5 minutes; 95% CI −23.5 to 14.5), or sr-MVPA per week (mean difference=3.8; 95% CI −1.9 to 9.5) at 3 months postrandomization or 6 months postrandomization (depressive symptoms: mean difference=−1.3; 95% CI −3.7 to 1.1; dm-MVPA: mean difference=1.3 minutes; 95% CI −18.9 to 21.5; sr-MVPA: mean difference=−1.8 MET-hours; 95% CI −7.7 to 4.2). Engagement with the intervention was suboptimal; although 52% (n=26) of participants allocated to the intervention group logged on to the intervention website and watched at least 1 video, the median minutes watched per week peaked at 10 minutes 2 weeks postrandomization, then fell to zero for the rest of the follow-up period. Results from modified intent-to-treat analyses were similar to those from intent-to-treat analyses.

**Conclusions:**

An eHealth PA intervention tailored to postpartum individuals did not affect depressive symptoms or PA among those at high risk for PPD. Additional research to develop effective and engaging PA interventions is needed to help alleviate PPD symptoms and decrease PPD risk.

## Introduction

Postpartum depression (PPD) is a debilitating and costly condition that affects nearly 20% of postpartum individuals [1,2]. PPD can have long-lasting health consequences for both postpartum individuals and their children, including negative maternal-infant interaction [3], lower likelihood of breastfeeding [4,5], and cognitive and behavioral problems [5-7]. Counseling is the currently recommended approach for prevention of PPD in individuals at high risk [8]; however, stigma and health care system barriers remain a challenge to accessing these mental health services for many postpartum individuals [9,10]. Alternative and complementary strategies are needed for PPD prevention .

There is strong evidence that physical activity (PA) can reduce PPD symptoms [11-15]. The PA Guidelines for Americans [16], the American College of Sports Medicine [17], and the American College of Obstetricians and Gynecologists [18] recommend participation in regular PA (at least 150 minutes of moderate-to-vigorous-intensity PA per week) during the postpartum period for physical and mental health benefits. Despite these recommendations, PA levels remain low in postpartum individuals [19]. Common barriers include lack of time and prioritization of other responsibilities, including parenting and household responsibilities, lack of child care, lack of motivation or social support, and difficulty finding accessible and affordable PA programs or classes [20-26].

Technology-based electronic health (eHealth) interventions may address common barriers to being physically active in the postpartum period, and as such are a promising approach to increasing PA levels in postpartum individuals at high risk for PPD. eHealth PA interventions have demonstrated effectiveness in increasing PA in the general adult population [27,28]. In contrast, most previous PA interventions for postpartum individuals have relied on in-person supervised exercise sessions alone or in combination with individual health coaching [13,29,30].

The objective of this study, the POstpartum Wellness study (POW), was to test the effectiveness of an eHealth PA intervention (MomZing) for increasing PA and decreasing depressive symptoms in individuals at high risk for PPD. MomZing is a web-based library of short, tailored exercise videos developed with and for postpartum individuals to address accessibility (no equipment needed, designed to be done at home), time constraints (10 min videos available on demand), and child care concerns (exercises are designed to incorporate baby as weight), common barriers to PA in postpartum.

## Methods

Below we summarize key aspects of the POW study protocol, which has been previously published [31].

### Study Design and Setting

We conducted a 2-arm, parallel-group randomized controlled trial among members of Kaiser Permanente Northern California (KPNC), an integrated health care delivery system that provides care for over 66,000 pregnant and postpartum individuals annually. PPD screening is part of standard postpartum care at KPNC [32,33] and also occurs at each pediatric well-baby visit. Results of all PPD screening and diagnosis are captured in KPNC’s comprehensive electronic health record database.

### Ethical Considerations

This study was approved by the KPNC Institutional Review Board (approval number 1548855) and registered with ClinicalTrials.gov on June 1, 2020 (ID: NCT04414696). All participants provided documented informed consent and could withdraw from the study at any time. All data are deidentified. Participants received gift cards as incentives after completing each assessment: US $20 after the baseline and 3-month assessments, and US $30 after the 6-month assessment (including the infant ASQ screener), for a total of US $70 in incentives.

### Recruitment and Eligibility

From November 2020 through September 2022, 12,269 postpartum individuals who met the following inclusion criteria in the electronic health record were invited to participate in the study via letter and email invitations: 2‐6 months postpartum, no current depression diagnosis, and at high risk for PPD (identified using PPD screening PHQ-9 score 10‐19 or PHQ-2 score ≥3, or history of depression diagnosis and antidepressant medication prescription prior to delivery date). Additional eligibility criteria (including reassessment of depression symptoms using a PHQ-8 screener) were assessed using online questionnaires prior to the baseline survey. See Table S1 in Multimedia Appendix 1 for detailed inclusion and exclusion criteria.

### Randomization and Blinding

Eligible participants completed informed consent documents and baseline surveys through the REDCap website (Research Electronic Data Capture; Vanderbilt University). After survey completion, participants were mailed an accelerometer and were asked to wear the accelerometer for 7 consecutive days, 24 hours per day, and then mail it back. Participants who returned the accelerometer were eligible to be randomized.

Participants were randomized by the study project manager into the intervention group or control (usual care) group using minimization, implemented using QMinim [34]. Minimization aims to achieve between-group balance in distributions of pre-specified important factors associated with outcomes of interest. Variables included in the randomization procedure included number of children at home (1, 2+), self-reported race and ethnicity category (Asian/Pacific Islander, Hispanic, non-Hispanic Black, non-Hispanic White, other), baseline PHQ-8 score (Patient Health Questionnaire-8; 10-14, 15-19), and PA level prior to pregnancy (below recommendations, at or above recommendations). Investigators and data analysts were blinded to the assigned study group. Research assistants, who informed participants of their assigned study group, and participants were not blinded.

### Intervention: MomZing

In addition to usual postpartum care, participants randomized to the intervention group received access to MomZing (Klein Buendel), an eHealth PA intervention tailored to postpartum individuals [35]. MomZing is an online library of 98 ten-minute exercise videos that was developed with and for postpartum individuals. The exercise videos on MomZing instructed postpartum individuals how to safely exercise with their baby based on the infant’s weight and developmental stage, did not require exercise equipment, and featured postpartum individuals (who were not fitness instructors) exercising with their own infant. The website also included a page to set a weekly PA goal and visually tracked (via a graph) the number of videos watched per week. Participants randomized to the intervention group received individual login information for the MomZing website via email and up to 6 reminders (text and email per each participant’s preference) to log into the MomZing website (73% text and 27% email).

### Control: Usual Care

Participants randomized to the control group received usual postpartum care for women at increased risk of depression, which typically is a brief discussion about their depression symptoms with their obstetric provider.

### Follow-Up Data Collection

Follow-up surveys were sent via email at 3 and 6 months postrandomization. After completion of each survey, participants were mailed an accelerometer and were asked to wear the accelerometer for 7 consecutive days, 24 hours per day, and then mail it back. An additional assessment occurred when the participant’s child was 12 months old; for some participants, this occurred simultaneously with the 6-month follow-up surveys. Follow-up data collection ended in April 2023.

### Primary Outcomes

Primary outcomes were depressive symptoms and device-measured moderate-to-vigorous intensity PA (dm-MVPA), measured at 3 months postrandomization. Depressive symptoms were assessed using the validated 8-item PHQ-8 screener [36,37], with higher scores indicating greater symptom severity (range 0‐24)*.* dm-MVPA was measured using the ActiGraph GT3X+ accelerometer (ActiGraph, Pensacola), worn on the non-dominant wrist. Raw accelerometer data were downloaded using ActiLife software (ActiGraph, Pensacola) and counts were summarized for each 1-second and 60-second epoch. Wear time was identified using the Choi algorithm [38-40], and wake (non-bedrest) was identified using the Tracy algorithm [41]. Individuals with at least 4 days (including at least 1 weekend day) of at least 10 hours of waking accelerometer wear were considered to have sufficient accelerometer data for analyses [42]. Waking wear time was categorized into intensity categories using the Hibbing 2-regression model, which was developed for wrist-worn accelerometer data [43]. Average dm-MVPA duration was calculated across valid days.

### Secondary Outcomes

Secondary outcomes included self-reported moderate-to-vigorous intensity PA (sr-MVPA) at 3 and 6 months postrandomization, and depressive symptoms and dm-MVPA at 6 months postrandomization. sr-MVPA duration was assessed using the sports and exercise domain of the Pregnancy Physical Activity Questionnaire [44]. In addition, we created a subscale of the sports and exercise domain of the Pregnancy Physical Activity Questionnaire (sr-MVPA-MomZing) limited to activities included in MomZing videos: dancing; yoga or Pilates or stretching or core strengthening exercises, exercise videos, or computer games; aerobic exercise videos or computer games; and weightlifting and resistance exercises (ie, MomZing included workouts where the infant was lifted or carried).

### Additional Outcomes

Additional outcomes were measured using validated questionnaires and included sleep quality, anxiety symptoms, and perceived stress at 3- and 6- months postrandomization, mother-infant bonding at 3 months postrandomization, and infant development at 12 months of age. Sleep quality was measured using the Pittsburgh Sleep Quality Index [45], with higher scores indicating worse sleep quality (range 0‐21). Anxiety symptoms were measured using the GAD-7 screener [46,47], with higher scores indicating greater symptom severity (range 0‐21). Perceived stress was measured using the Perceived Stress Scale [48,49], with higher scores indicating higher stress (range 0‐40). Mother-infant bonding was assessed using the Mother Infant Bonding Scale [50], with lower scores indicating better bonding (range 0‐24). Infant development across 5 domains (communication, gross motor function, fine motor function, problem-solving, and personal social skills) was assessed using the Ages and Stages Questionnaire-3 for 12-month-old infants [51], with higher scores indicating more typical (“on schedule”) development (range 0‐60).

### Adverse Event Reporting

Participants reported injuries or illnesses related to exercise at 3 and 6 months postrandomization.

### Participant Feedback About the Intervention

Participants allocated to the intervention group received a feedback survey at 3 months postrandomization that assessed their use of the MomZing website, reasons for not using the website, experience and satisfaction with the MomZing intervention, and qualitative feedback about the intervention. The satisfaction questions were adapted from a validated satisfaction questionnaire for web-based health interventions with Likert scale responses, with higher scores indicating higher satisfaction (range 12‐60) [52].

### Sample Size Determination

Original sample size calculations were based on a planned sample size of 100 participants per group. With our achieved sample size of 99 participants (50 in the intervention group, 49 in the control group), 80% power, and 5% type I error rate, the minimum detectable difference in means for depressive symptoms and dm-MVPA was 0.57 standard deviation units, which is considered a “medium” effect size [53]. Using the observed outcome distributions (Table 1), this corresponds to 3.2 units for PHQ-8 score and 24.1 minutes for dm-MVPA.

**Table 1.** Effect of the MomZing intervention on primary outcomes (measured at 3 months postrandomization).

	Control Mean (SD)	Intervention Mean (SD)	Adjusted mean difference (95% CI)^a^
Intent-to-treat analysis			
Depressive symptoms	11 (5.8)	10.1 (5.4)	−0.9 (-3.3 to 1.5)
dm-MVPA^b^ (minutes per day)	58 (44.8)	53.2 (39.9)	−4.5 (−23.5 to 14.5)
Modified intent-to-treat analysis			
Depressive symptoms	11 (5.8)	10 (5.8)	−0.8 (−3.9 to 2.3)
dm-MVPA (minutes per day)	58 (44.8)	52.2 (38.2)	−0.5 (−23.2 to 22.2)

a Adjusted for baseline PHQ-8 (Patient Health Questionnaire-8) score, race and ethnicity, number of children at home, pre-pregnancy PA level, and corresponding baseline outcome measure

b dm-MVPA, device-measured moderate-to-vigorous intensity physical activity.

### Statistical Analysis

We compared baseline characteristics between the intervention and control groups and between the entire intervention group and the subset of the intervention group who engaged with the intervention (watched at least 1 MomZing video).

Primary analyses followed an intent-to-treat approach. We used multivariable linear regression models, adjusted for variables used in the randomization procedure (number of children at home, race and ethnicity, baseline PHQ-8 score, pre-pregnancy PA level) and corresponding baseline value of the outcome, to estimate mean differences in each outcome associated with the MomZing intervention.

Secondary analyses followed a modified intent-to-treat approach. We excluded participants allocated to the intervention group who did not engage with the intervention (did not watch at least 1 video on the MomZing website during the 3-month period postrandomization).

We used multiple imputation using chained equations to impute missing outcome and baseline MVPA data with 10 imputations. Variables included in imputation models are shown in Table S2 in Multimedia Appendix 1. Regression models were run using each imputed dataset. Results were combined using Rubin’s rules [54]. We also conducted complete case analysis as a sensitivity analysis. Analyses were conducted in SAS (version 9.4; SAS Institute Inc.) and R (version 4.3.1; R Foundation for Statistical Computing).

## Results

### Participant Characteristics

**Figure 1.**
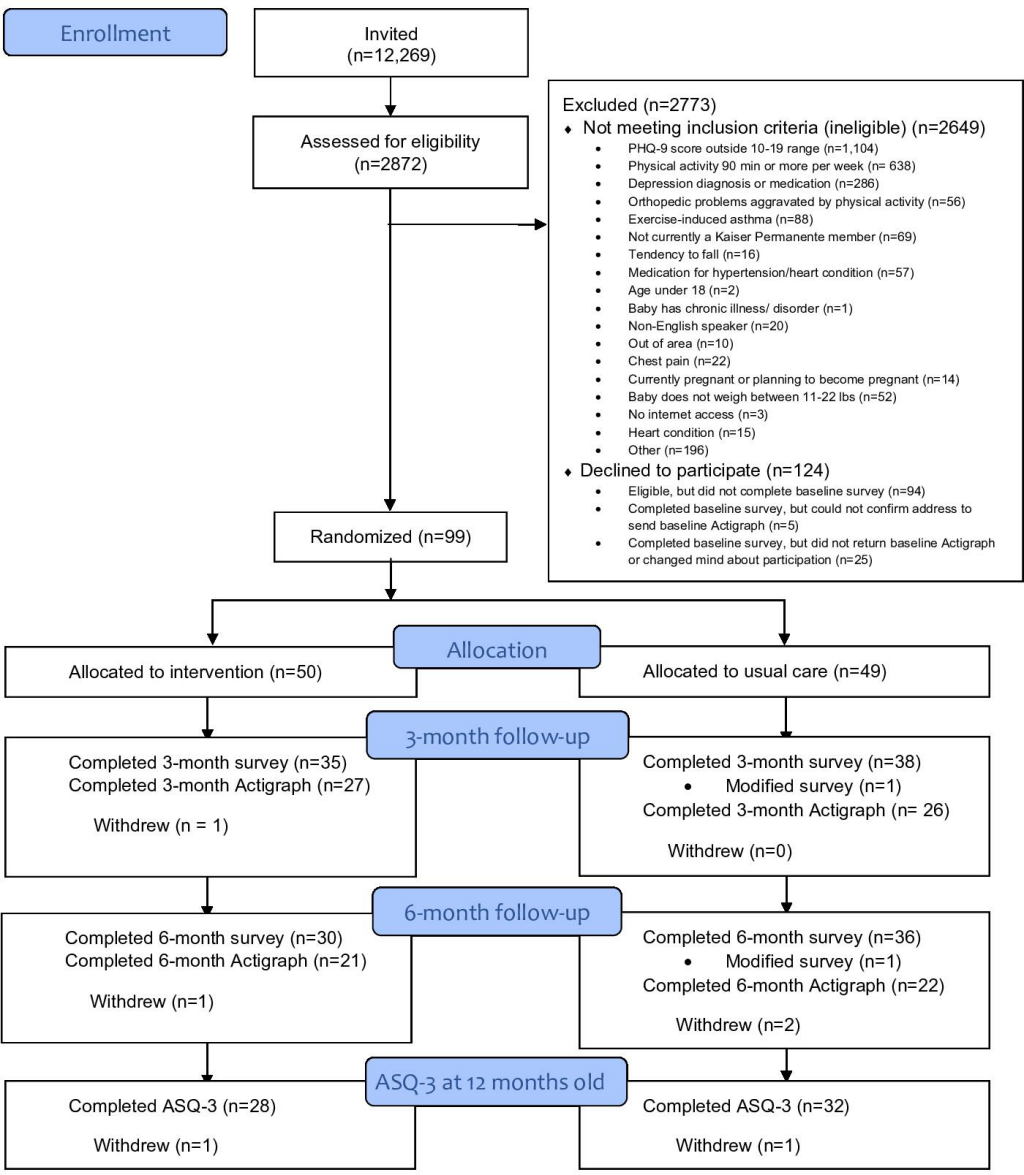
CONSORT flow diagram. ASQ-3: Ages and Stages Questionnaire-3; PHQ: Patient Health Questionnaire.

Of the 99 randomized participants, 27 (54%) and 26 (53%) in the intervention and control groups, respectively, completed all 3-month follow-up activities (online surveys and accelerometer; Figure 1). At baseline, participants were 4 months postpartum, on average, and had moderately severe depressive symptoms (mean PHQ-8=12.6; Table 2). Most participants were members of a minoritized racial or ethnic group (63%), not currently working (74%), married or living with their partner (86%), did not meet PA recommendations prior to pregnancy (61%), had a college education or more (80%), and had 2 or more children at home (59%). Baseline characteristics were similar between the intervention and control groups (Table 2).

**Figure 2.**
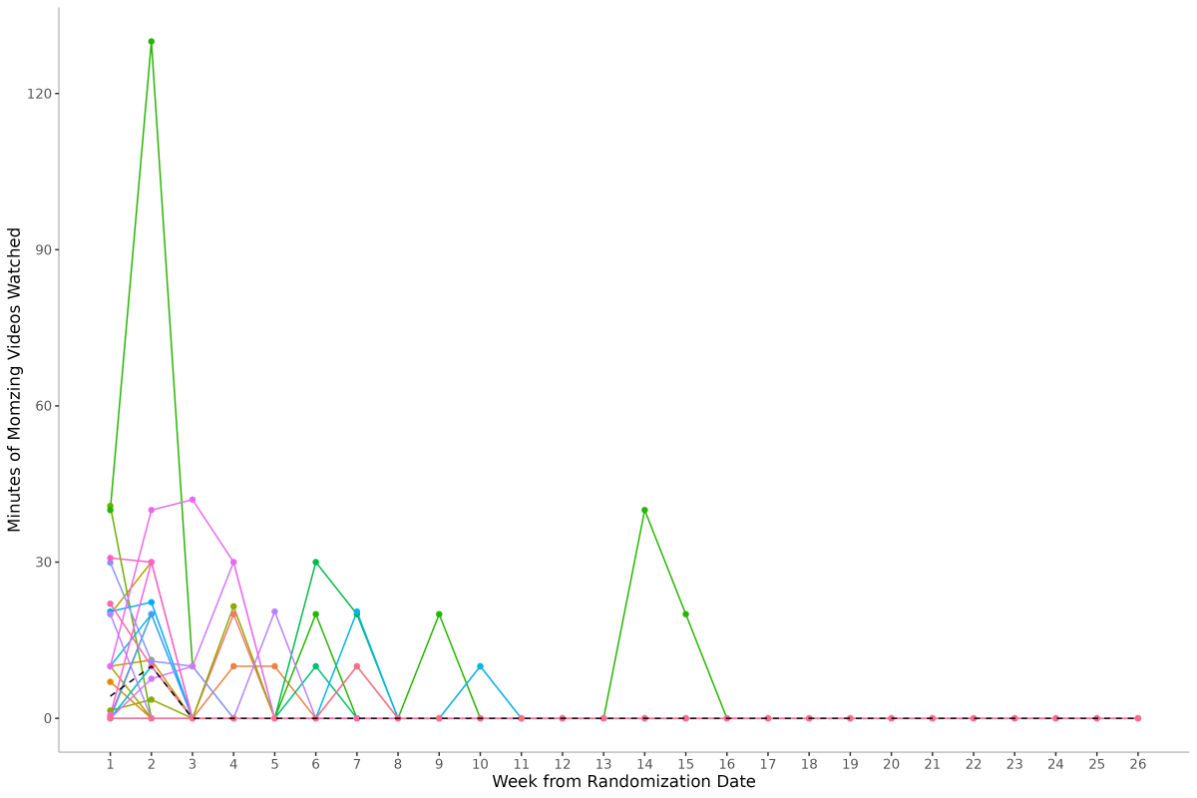
Minutes of MomZing videos watched per week among intervention participants who logged in to MomZing and watched at least 1 video (n=26). Each color represents 1 participant. The black dotted line indicates median minutes per week.

In total, 52% (n=26) of participants allocated to the intervention group logged in to the MomZing website and watched at least 1 video. The median minutes of videos watched per week peaked at 10 minutes 2 weeks after randomization, then fell to zero for the rest of the follow-up period (Figure 2). Baseline characteristics were similar between participants who engaged with the intervention (watched at least 1 MomZing video) and participants in the control group . Within those allocated to the intervention, characteristics differed somewhat by engagement with the intervention: compared to participants who did not engage with the intervention, those who did were fewer months postpartum, more likely to be non-Hispanic White, more likely to have a household income of US $100,000 or greater per year, less likely to be working, and less likely to meet PA recommendations in pre-pregnancy (Table S3 in Multimedia Appendix 1).

**Table 2.** Baseline participant characteristics by study group.

Characteristics	Control n=49	Intervention n=50
Age (years), mean (SD)	32.7 (4.5)	31.9 (5.1)
Months postpartum, mean (SD)	4 (1)	4.1 (1.2)
Race and ethnicity, n (%)		
Asian/Pacific Islander	4 (8.2)	6 (12)
Hispanic	12 (24.5)	10 (20)
Non-Hispanic Black	3 (6.1)	3 (6)
Non-Hispanic White	18 (36.7)	19 (38)
Other^a^	12 (24.5)	12 (24)
Highest level of education, n (%)		
College	29 (59.2)	31 (62)
Graduate school	12 (24.5)	7 (14)
High school or less	8 (16.3)	11 (22)
Unknown	0 (0)	1 (2)
Annual household income, n (%)		
US $100,000 or greater per year	23 (46.9)	17 (34)
US $65,000 to $99,999 per year	9 (18.4)	14 (28)
Less than US $65,000 per year	13 (26.5)	13 (26)
Unknown	4 (8.2)	6 (12)
Employment status, n (%)		
Not currently working	34 (69.4)	39 (78)
Currently working	15 (30.6)	11 (22)
Marital status, n (%)		
Married or living with partner	44 (89.8)	41 (82)
Single or divorced	5 (10.2)	9 (18)
Number of children at home, n (%)		
2+	28 (57.1)	30 (60)
1	21 (42.9)	20 (40)
Pre-pregnancy PA level, n (%)		
Below recommendations	31 (63.3)	29 (58)
At or above recommendations	18 (36.7)	21 (42)
sr-MVPA^b^ (MET^c^-hours per week), mean (SD)	7.4 (15.8)	6.5 (9)
Unknown	1	0
sr-MVPA-MomZing (MET-hours per week), mean (SD)	0.9 (1.9)	1.9 (3.9)
Unknown	1	0
dm-MVPA^d^ (minutes per day), mean (SD)	49.9 (45.8)	48.3 (32.7)
Unknown	4	3
Mental health and bonding		
Depressive symptoms, mean (SD)	12.6 (2.2)	12.6 (2.2)
Anxiety symptoms, mean (SD)	11.7 (4.6)	12.4 (5.1)
Perceived stress, mean (SD)	25.2 (5.6)	24.4 (5.5)
Mother-infant bonding, mean (SD)	2 (2.5)	1.7 (2.5)

a Other includes Multiracial, Native American, and unknown.

b sr-MVPA: self-reported moderate-to-vigorous intensity PA.

c MET: metabolic equivalent of task.

d dm-MVPA: device-measured moderate-to-vigorous intensity PA.

### Intent-to-Treat Analyses

#### Primary Outcomes

There was no association between the MomZing intervention and change in depressive symptoms (mean difference=−0.9; 95% CI −3.3 to 1.5) or dm-MVPA per day (mean difference=−4.5 min; 95% CI −23.5 to 14.5) at 3 months postrandomization (Table 1).

#### Secondary outcomes

As shown in Table 3, there were no differences between groups in sr-MVPA or sr-MVPA-MomZing per week at 3 months postrandomization and no differences between groups in change in depressive symptoms, dm-MVPA per day, and sr-MVPA or sr-MVPA-MomZing per week at 6 months postrandomization.

**Table 3.** Effect of the MomZing intervention on secondary outcomes.

	Control Mean (SD)	Intervention Mean (SD)	Adjusted mean difference (95% CI)^a^
Intent-to-treat analysis			
3 months postrandomization			
sr-MVPA^b^ (MET^c^ hours per week)	12.7 (14.7)	15.8 (15.7)	3.8 (−1.9 to 9.5)
sr-MVPA-MomZing (MET hours per week)	2.2 (3.2)	4.7 (5.8)	1.9 (−0.1 to 3.9)
6 months postrandomization			
Depressive symptoms	9.1 (5.4)	7.9 (4.9)	−1.3 (−3.7, to1.1)
dm-MVPA^d^ (minutes per day)	62.3 (53.8)	63.2 (49.1)	1.3 (−18.9 to 21.5)
sr-MVPA (MET hours per week)	16.3 (16.6)	13.7 (13.8)	−1.8 (−7.7 to 4.2)
sr-MVPA-MomZing (MET hours per week)	2.5 (4.6)	3 (3.9)	0.1 (−1.8 to 2.1)
Modified intent-to-treat analysis			
3 months postrandomization			
sr-MVPA (MET hours per week)	12.7 (14.7)	15 (15)	3.3 (−3.3 to10)
sr-MVPA-MomZing (MET hours per week)	2.2 (3.2)	5.6 (7)	2.8 (0.2 to 5.4)
6 months postrandomization			
Depressive symptoms	9.1 (5.4)	8.3 (5.1)	−0.7 (−3.8 to 2.5)
dm-MVPA (minutes per day)	62.3 (53.8)	58.9 (45.5)	2.5 (−21.1 to 26.1)
sr-MVPA (MET hours per week)	16.3 (16.6)	11.2 (12.3)	−4.9 (−12.6 to 2.8)
sr-MVPA-MomZing (MET hours per week)	2.5 (4.6)	2.7 (3.8)	−0.6 (−3 to 1.8)

a Adjusted for baseline PHQ-8 (Patient Health Questionnaire-8) score, race and ethnicity, number of children at home, pre-pregnancy physical activity (PA) level, and corresponding baseline outcome measure.

b sr-MVPA: self-reported moderate or vigorous intensity PA.

c MET: metabolic equivalent of task.

d dm-MVPA: device-measured moderate or vigorous intensity PA.

#### Additional Outcomes

As shown in Table 4, the MomZing intervention was not associated with change in sleep quality, anxiety symptoms, or perceived stress at 3 or 6 months postrandomization, mother-infant bonding score at 3 months postrandomization, or infant development in any area.

**Table 4.** Effect of the MomZing intervention on additional mental health, bonding, and infant development outcomes

	Control Mean (SD)	Intervention Mean (SD)	Adjusted mean difference (95% CI)^a^
Intent-to-treat analysis			
3 months postrandomization			
Sleep quality	10.1 (4.2)	9.6 (4)	0.5 (−1.2 to 2.1)
Anxiety symptoms	10 (5.7)	9.2 (5.4)	−1.1 (−3.4 to 1.2)
Perceived stress	23.2 (7.3)	21 (6.7)	−1.8 (−4.9 to 1.3)
Mother-infant bonding	1.8 (3)	1.7 (2.9)	0 (−1 to 1.1)
6 months postrandomization			
Sleep quality	8.7 (3.9)	8.7 (3.4)	0.5 (−1 to 2.3)
Anxiety symptoms	8.4 (5.2)	8 (4.8)	−0.5 (−2.8 to 1.8)
Perceived stress	20.9 (5.5)	19.5 (5.2)	−1.2 (−3.7 to 1.2)
Infant development at 12 months of age			
Communication	42.1 (14.7)	44.6 (13.9)	2.5 (−4.9 to 9.9)
Gross motor function	47.5 (14.5)	47.6 (15.1)	0.2 (−7 to 7.4)
Fine motor function	48.5 (9.5)	50 (9.4)	1.7 (−2.8 to 6.2)
Problem-solving	41.1 (13.8)	42 (12.9)	1.2 (−4.4 to 6.9)
Personal-social skills	36.9 (14)	40.1 (13.5)	3.6 (−2.9 to10)
Modified intent-to-treat analysis			
3 months postrandomization			
Sleep quality	10.1 (4.2)	9.9 (4.1)	0.7 (−1.6 to 2.8)
Anxiety symptoms	10 (5.7)	9.6 (5.5)	−0.2 (−2.9 to 2.6)
Perceived stress	23.2 (7.3)	21.5 (7.2)	−1.3 (-−5.3 to 2.7)
Mother-infant bonding	1.8 (3)	1.8 (2.4)	0.1 (−1.2 to 1.3)
6 months postrandomization			
Sleep quality	8.7 (3.9)	8.4 (3.4)	0.3 (−1.6 to 2.1)
Anxiety symptoms	8.4 (5.2)	7.9 (5.1)	−0.1 (−3.1 to 2.8)
Perceived stress	20.9 (5.5)	20.4 (5.2)	−0.2 (−3.2 to 2.9)
Infant development at 12 months of age			
Communication	42.1 (14.7)	42.9 (14.4)	1.2 (−7.6 to 9.9)
Gross motor function	47.5 (14.5)	44.8 (16.3)	−2.6 (−11.4 to 6.3)
Fine motor function	48.5 (9.5)	48.4 (10.2)	0.7 (−4.6 to 6)
Problem-solving	41.1 (13.8)	39.8 (13.4)	0.1 (−7.7 to 7.9)
Personal social skills	36.9 (14)	37.9 (14.6)	2.2 (−6.6 to 11)

a Adjusted for baseline PHQ-8 (Patient Health Questionnaire-8) score, race and ethnicity, number of children at home, pre-pregnancy physical activity level, and corresponding baseline outcome measure.

### Modified Intent-to-Treat Analyses

Findings from modified intent-to-treat analyses were similar to those from intent-to-treat analyses for primary, secondary, and additional outcomes (Tables1, 3  4).

### Sensitivity Analyses

Findings from complete case analyses were similar to those using imputation (data not shown).

### Adverse Events

There were no adverse events reported during the study.

### Participant Feedback About the Intervention

Among those who reported using the MomZing website (44%, n=22), the mean satisfaction score was 40, indicating moderately high overall satisfaction with the intervention (Table 5). In total, 90% rated MomZing as good or very good or excellent. Over 60% of participants who reported using the MomZing website found it very or moderately helpful for being more physically active. Qualitative feedback also indicated that participants wanted more frequent reminders to use the MomZing website, more intense workouts, and an app that is accessible on multiple devices. The most common reason for not using the MomZing website was lack of time (77%).

**Table 5.** Participant evaluation of the MomZing intervention 3 months postrandomization.

Feedback item	Value
Satisfaction and experience with MomZing^a^ (n=22)	
Satisfaction score, mean (SD)	40 (9)
Overall rating of MomZing, n (%)	
Excellent or very good	10 (45%)
Good	10 (45%)
Fair or poor	2 (9%)
How helpful did you find the MomZing workout videos for being more physically active?, n (%)	
Very or moderately helpful	15 (68%)
Of little help or not helpful	7 (32%)
Reasons for not using the MomZing website^b^ (n=13), n (%)	
Did not have time	10 (77%)
Was not sure what my login information was	2 (15%)
Did not find the website helpful	2 (15%)
Other: Used other workout program or walked instead	2 (15%)
Did not understand how to use it	1 (8%)
Was not interested in using the website	1 (8%)
Did not have access to the internet	0
Did not like the website	0

a Limited to respondents who reported using the MomZing website.

b Limited to respondents who reported not using the MomZing website.

## Discussion

In this 2-arm, parallel-group randomized controlled trial, there were no statistically significant differences in change in depressive symptoms or dm-MVPA over a 3-month period associated with the MomZing eHealth PA intervention in postpartum individuals at high risk of PPD. There were also no differences in change in sr-MVPA, sleep quality, anxiety symptoms, perceived stress, mother-infant bonding, or infant development at 12 months of age associated with the intervention.

Engagement with the intervention was low, with roughly half of intervention group participants logging into the MomZing website and peak weekly engagement of a median of 10 minutes of videos watched at 2 weeks after randomization, which may explain our null findings in both intent-to-treat and modified intent-to-treat analyses. Participants in the intervention group who used the MomZing website rated it highly; however, those who did not use the MomZing website reported that lack of time remained a barrier to PA. Additionally, for some participants the MomZing intervention did not meet their expectations for quality and content, as they preferred higher intensity workouts and accessibility of content via an app. A previous review of behavior change techniques present in efficacious PA interventions among postpartum populations identified goal setting for PA and self-monitoring of PA as important components (ie, these techniques were present in all efficacious interventions) [55]. While the MomZing website did allow for setting a weekly PA goal and visually tracked the number of videos watched per week, there was no promotion of these activities in our study materials, which may also have contributed to our null findings. Providing goal-setting and self-monitoring tools without additional social support is likely not enough to change PA behavior in this population, as previous research suggests that social support is the strongest contributor to PA behavior change in postpartum individuals [56].

Previous randomized controlled trials of eHealth PA interventions in postpartum populations have used various eHealth modalities, including a mobile app alone [57] or paired with free exercise equipment for the home [58], a Facebook group [59], and an exercise DVD [60]; only 1 study was conducted among individuals at high risk of PPD [58], and all reported no associations with mental health outcomes despite higher intervention engagement and retention compared to the present study. Teychenne et al [58] pilot tested whether a 12-week home-based PA intervention, which included a free in-home treadmill or stationary bike, mobile app, and online forum, changed PA, sleep, and mental health in postpartum individuals at high risk for PPD (n=62). Results showed that the intervention group had less dm-MVPA compared to the control group (−1.3% of the day), and there were no differences in sr-MVPA, depressive symptoms, anxiety symptoms, or sleep quality. Other studies have been conducted among the general population of postpartum individuals. In a pilot study with 35 participants, Tinius et al [57] did not find differences in dm-MVPA or sr-MVPA at 12 weeks postpartum between participants in an attention control group and those who had access to a mobile app to help perinatal individuals (mid-pregnancy to postpartum) meet PA recommendations, which included PA tracking, exercise videos, and educational content. In a trial with 120 participants, Kernot et al [59] did not find differences in dm-MVPA or self-reported walking between participants in a 50-day team-based PA intervention delivered via Facebook and participants in a control group. Finally, Yang et al [61] did not report differences in depressive symptoms, sleep quality, or perceived stress in a pilot study (n=140) of a 12-week aerobic gymnastic intervention designed for postpartum individuals and delivered via DVD. These findings, along with our study results, highlight the need for additional research to develop effective PA interventions for postpartum individuals at high risk of PPD and for the postpartum population in general.

Strengths of this study include using an intervention tailored for postpartum individuals that was developed with key stakeholder input; assessment of dm-MVPA and sr-MVPA; assessment of multiple aspects of mental health; multiple timepoints of outcome assessment; inclusion of participants from a large population sample; and use of statistical techniques for balancing groups. However, several limitations should be noted, which highlight the challenges in PA and research engagement during postpartum, especially in those at high risk for PPD and may explain our null results. We did not meet our recruitment goal, and as a result, our study is underpowered for effect sizes smaller than “medium,” resulting in imprecise estimates of associations (wide confidence intervals). As discussed previously, engagement with the intervention was low, which can lead to underestimation of intervention effects in intent-to-treat analyses [62] and further reduces sample size and power in modified intent-to-treat analyses. Study recruitment occurred from November 2020 to September 2022, during the COVID-19 pandemic when many stressors and barriers to PA were heightened [63], which may have reduced motivation for and interest in PA. This was also when many other online PA offerings were becoming available, which may have been more attractive to participants, resulting in low engagement with the MomZing intervention and highlighting potential needs for improvement in production value and content of the website. Finally, retention for follow-up assessments was a challenge, likewise potentially due in part to the challenges of the COVID-19 pandemic; however, multiple imputation mitigated this limitation.

In conclusion, an eHealth PA intervention tailored to postpartum individuals did not change PA, mental health, sleep quality, perceived stress, mother-infant bonding, or infant development among those at high risk for PPD. Additional research, including qualitative data, to develop effective and engaging PA interventions for this population is needed to help alleviate PPD symptoms and decrease PPD risk.

## Supplementary material

10.2196/64507 Multimedia Appendix 1 Supplemental tables.

10.2196/64507 Checklist 1 CONSORT-EHEALTH checklist (V 1.6.1).

## Abbreviations

dm-MVPA: device-measured moderate or vigorous intensity PA
eHealth: electronic health
KPNC: Kaiser Permanente Northern California
POW: Postpartum Wellness study
PPD: postpartum depression
sr-MVPA: secondary outcomes were self-reported MVPA
