# Supplementary material for: Effect of a Tailored eHealth Physical Activity Intervention on Physical Activity and Depression During Postpartum: Randomized Controlled Trial (The Postpartum Wellness Study)
Source: JMIR Ment Health. 2025 May 22;12:e64507. doi: 10.2196/64507 (PMC12121695; doi:10.2196/64507)
Supplement: Multimedia Appendix 1 [file mental-v12-e64507-s001.docx]

SUPPLEMENTAL MATERIAL

**Effect of a Tailored eHealth Physical Activity Intervention on Physical Activity and Depression during Postpartum: The POstpartum Wellness Study (POW), a Randomized Controlled Trial among Individuals at High Risk of Postpartum Depression**

Table S1. Inclusion and exclusion criteria for the Postpartum Wellness (POW) randomized controlled trial

Table S2. Variables included in imputation models

Table S3. Baseline participant characteristics by intervention engagement

| Table S1. Inclusion and exclusion criteria for the Postpartum Wellness (POW) randomized controlled trial | |
| --- | --- |
| **Identification of potentially eligible participants** via the  Electronic Health Record (EHR) | - 2-6 months postpartum - No current depression diagnosis - Postpartum PHQ-9 score between 10-19 OR postpartum PHQ-2 score ≥3 OR history of depression diagnosis or antidepressant medication use |
| **Inclusion criteria**  Ascertained via eligibility screener | - PHQ-8 score between 10-19 - Engages in <90 minutes of regular, moderate/vigorous intensity physical activity per week |
| **Exclusion Criteria**  Ascertained via eligibility screener | - Not a current Kaiser Permanente member - < 18 years of age - Does not own a smartphone, computer, or TV with internet access - Has a heart condition and a physician recommending medically supervised physical activity - Has chest pain during physical activity or chest pain within the prior month - Takes medication for hypertension or a heart condition - Diagnosed with depression or received treatment for depression (e.g., taken antidepressant medications or received psychotherapy) since giving birth - Tendency to fall due to syncope or dizziness - Has orthopedic problems that might be aggravated by physical activity - Has exercise-induced asthma - Is currently pregnant or is planning to become pregnant in the next three months - Baby weighs outside the range of 11-22 lbs - Baby has a chronic illness/disorder(s) that prevent them from being held or lifted up |

| Table S2. Variables included in imputation models | | |
| --- | --- | --- |
| **Variable** | **Timepoint(s)** | **Included for outcomes** |
| Randomization group | N/A | All |
| Any MomZing videos watched (yes/no) | N/A | All |
| Number of children living at home | Baseline | All |
| Pre-pregnancy PA level | Baseline | All |
| Race and ethnicity  (one variable) | Baseline | All |
| Age | Baseline | All |
| Highest level of education | Baseline | All |
| Annual household income | Baseline | All |
| Number of people supported by household income | Baseline | All |
| PHQ-8 score | Baseline, 3 months, 6 months | PHQ-8, dm-MVPA |
| sr-MVPA  (MET-hours per week) | Baseline, 3 months, 6 months | PHQ-8, dm-MVPA, sr-MVPA, sr-MVPA-MomZing |
| sr-MVPA-MomZing  (MET-hours per week) | Baseline, 3 months, 6 months | PHQ-8, dm-MVPA, sr-MVPA, sr-MVPA-MomZing |
| GAD-7 score | Baseline, 3 months | GAD-7, PSQI, PSS-10, MIBS, ASQ |
| PSS-10 score | Baseline, 3 months, 6 months | GAD-7, PSQI, PSS-10, MIBS, ASQ |
| MIBS score | Baseline, 3 months | GAD-7, PSQI, PSS-10, MIBS, ASQ |
| PSQI score | Baseline, 3 months, 6 months | GAD-7, PSQI, PSS-10, MIBS, ASQ |
| dm-MVPA (average minutes per day) | Baseline, 3 months, 6 months | PHQ-8, dm-MVPA |
| ASQ scores | 12 months | GAD-7, PSQI, PSS-10, MIBS, ASQ |
| ASQ, Ages and Stages Questionnaire, Third Edition; dm-, device-measured; GAD-7, 7-Item Generalized Anxiety Disorder Scale; MIBS, Mother Infant Bonding Scale; MVPA, moderate/vigorous intensity physical activity; PA, physical activity; PHQ-8, 8-item Patient Health Questionnaire depression scale; PSQI, Pittsburgh Sleep Quality Index; PSS-10, 10-Item Perceived Stress Scale; sr-, self-reported | | |

| Table S3. Baseline participant characteristics by intervention engagement | | |
| --- | --- | --- |
|  | **Engaged with Intervention**  n=26 | **Did not Engage with Intervention**  n=24 |
| **Sociodemographic characteristics** | | |
| Age (years), mean (SD) | 33.2 (4.4) | 30.5 (5.4) |
| Months postpartum, mean (SD) | 3.7 (0.8) | 4.7 (1.3) |
| Race and ethnicity, n (%) |  |  |
| Asian/Pacific Islander | 3 (11.5) | 3 (12.5) |
| Hispanic | 2 (7.7) | 8 (33.3) |
| Non-Hispanic Black | 3 (11.5) | 0 (0.0) |
| Non-Hispanic White | 14 (53.8) | 5 (20.8) |
| Other^a^ | 4 (15.4) | 8 (33.3) |
| Highest level of education, n (%) |  |  |
| College | 16 (61.5) | 15 (62.5) |
| Graduate School | 5 (19.2) | 2 (8.3) |
| High School or less | 5 (19.2) | 6 (25.0) |
| Unknown | 0 (0.0) | 1 (4.2) |
| Annual household income, n (%) |  |  |
| $100,000 or greater per year | 12 (46.2) | 5 (20.8) |
| $65,000 to $99,999 per year | 9 (34.6) | 5 (20.8) |
| Less than $65,000 per year | 2 (7.7) | 11 (45.8) |
| Unknown | 3 (11.5) | 3 (12.5) |
| Employment status, n (%) |  |  |
| Not currently working | 22 (84.6) | 7 (29.2) |
| Currently working | 4 (15.4) | 17 (70.8) |
| Marital status, n (%) |  |  |
| Married/Living with Partner | 21 (80.8) | 20 (83.3) |
| Single/Divorced | 5 (19.2) | 4 (16.7) |
| Number of children at home, n (%) |  |  |
| 2+ | 17 (65.4) | 13 (54.2) |
| 1 | 9 (34.6) | 11 (45.8) |
|  |  |  |
| **Physical activity** | | |
| Pre-pregnancy PA level, n (%) |  |  |
| Below recommendations | 14 (53.8) | 9 (37.5) |
| At or above recommendations | 12 (46.2) | 15 (62.5) |
| sr-MVPA (MET-hours per week), mean (SD) | 6.7 (8.4) | 6.3 (9.7) |
| sr-MVPA-MomZing (MET-hours per week), mean (SD) | 2.4 (4.9) | 1.3 (2.4) |
| dm-MVPA (minutes per day), mean (SD) | 38.8 (23.6) | 59.0 (38.5) |
| Unknown | 1 | 2 |
|  |  |  |
| **Mental health and bonding** | | |
| Depressive symptoms, mean (SD) | 12.5 (1.8) | 12.7 (2.5) |
| Anxiety symptoms, mean (SD) | 11.5 (4.9) | 13.3 (5.1) |
| Perceived stress, mean (SD) | 24.2 (5.3) | 24.6 (5.8) |
| Mother-infant bonding, mean (SD) | 2.0 (2.8) | 1.4 (2.3) |
| dm-, device-measured; MET, Metabolic Equivalent of Task; MVPA, moderate/vigorous intensity physical activity; PA, physical activity; SD, standard deviation; sr-, self-reported  ^a^Other includes Multiracial, Native American, & unknown | | |
